# Epidemiology and Clinical Management of *Fusarium* keratitis in the Netherlands, 2005–2016

**DOI:** 10.3389/fcimb.2020.00133

**Published:** 2020-04-03

**Authors:** Claudy Oliveira dos Santos, Eva Kolwijck, Jeroen van Rooij, Remco Stoutenbeek, Nienke Visser, Yanny Y. Cheng, Nathalie T. Y. Santana, Paul E. Verweij, Cathrien A. Eggink

**Affiliations:** ^1^Department of Medical Microbiology, Radboud University Medical Center, Nijmegen, Netherlands; ^2^Centre of Expertise in Mycology Radboudumc/CWZ, Nijmegen, Netherlands; ^3^Department of Medical Microbiology, University Medical Center Groningen, University of Groningen, Groningen, Netherlands; ^4^Rotterdam Eye Hospital, Rotterdam, Netherlands; ^5^Department of Ophthalmology, University Medical Center Groningen, University of Groningen, Groningen, Netherlands; ^6^Maastricht University Medical Center+, University Eye Clinic, Maastricht, Netherlands; ^7^Department of Ophthalmology, Leiden University Medical Center, Leiden, Netherlands; ^8^Department of Ophthalmology, Amsterdam University Medical Center, Amsterdam, Netherlands; ^9^Department of Ophthalmology, Radboud University Medical Center, Nijmegen, Netherlands

**Keywords:** fungal keratitis, *Fusarium*, susceptibility, identification, contact lenses, visual outcome, chlorhexidine

## Abstract

**Introduction:** Recognizing fungal keratitis based on the clinical presentation is challenging. Topical therapy may be initiated with antibacterial agents and corticosteroids, thus delaying the fungal diagnosis. As a consequence, the fungal infection may progress ultimately leading to more severe infection and blindness. We noticed an increase of fungal keratitis cases in the Netherlands, especially caused by *Fusarium* species, which prompted us to conduct a retrospective cohort study, aiming to describe the epidemiology, clinical management, and outcome.

**Materials and Methods:** As fungi are commonly sent to the Dutch mycology reference laboratory for identification and *in vitro* susceptibility testing, the fungal culture collection was searched for *Fusarium* isolates from corneal scrapings, corneal swabs, and from contact lens (CL) fluid, between 2005 and 2016. All *Fusarium* isolates had been identified up to species level through sequencing of the ITS1-5.8S-ITS2 region of the rDNA and TEF1 gene. Antifungal susceptibility testing was performed according to the EUCAST microbroth dilution reference method. Antifungal agents tested included amphotericin B, voriconazole, and natamycin. In addition, susceptibility to the antisepticum chlorhexidine was tested. Ophthalmologists were approached to provide demographic and clinical data of patients identified through a positive culture.

**Results:** Between 2005 and 2016, 89 cases of *Fusarium* keratitis from 16 different hospitals were identified. The number of cases of *Fusarium* keratitis showed a significant increase over time (*R*^2^ = 0.9199), with one case in the first 5 years (2005–2009) and multiple cases from 2010 and onwards. The male to female ratio was 1:3 (*p* = 0.014). Voriconazole was the most frequently used antifungal agent, but treatment strategies differed greatly between cases including five patients that were treated with chlorhexidine 0.02% monotherapy. Keratitis management was not successful in 27 (30%) patients, with 20 (22%) patients requiring corneal transplantation and seven (8%) requiring enucleation or evisceration. The mean visual acuity (VA) was moderately impaired with a logMAR of 0.8 (95% CI 0.6–1, Snellen equivalent 0.16) at the time of *Fusarium* culture. Final average VA was within the range of normal vision [logMAR 0.2 (95% CI 0.1–0.3), Snellen equivalent 0.63]. CL wear was reported in 92.9% of patients with *Fusarium* keratitis. The time between start of symptoms and diagnosis of fungal keratitis was significantly longer in patients with poor outcome as opposed to those with (partially) restored vision; 22 vs. 15 days, respectively (mean, *p* = 0.024). Enucleation/evisceration occurred in patients with delayed fungal diagnosis of more than 14 days after initial presentation of symptoms. The most frequently isolated species was *F. oxysporum* (24.7%) followed by *F. solani sensu stricto* (18%) and *F. petroliphilum* (9%). The lowest MICs were obtained with amphotericin B followed by natamycin, voriconazole, and chlorhexidine.

**Conclusion:** Although *Fusarium* keratitis remains a rare complication of CL wear, we found a significant increase of cases in the Netherlands. The course of infection may be severe and fungal diagnosis was often delayed. Antifungal treatment strategies varied widely and the treatment failure rate was high, requiring transplantation or even enucleation. Our study underscores the need for systematic surveillance of fungal keratitis and a consensus management protocol.

## Introduction

Fungal keratitis is a common eye infection in tropical and subtropical areas of the world, but is rarely observed in temperate climates. The clinical diagnosis of fungal keratitis is difficult, and antifungal therapy may be delayed due to primary therapy with antibacterial agents, antiviral agents and topical corticosteroids. As a consequence, the infection may progress in the cornea which ultimately may lead to more severe infection and monocular blindness. Although many fungi have been reported to cause fungal keratitis, *Fusarium* species are the most frequent cause (Gopinathan et al., 2002; Iyer et al., 2006). *Fusarium* species are fast-growing hyalohyphomycetes and are ubiquitous organisms that are present in soil, water, and plants. As filamentous fungi cannot penetrate intact cornea, the most common route of infection is through (micro) trauma or disruptive ocular surface disease. Several epidemiological studies indicate that the frequency of *Fusarium* keratitis may be increasing (Cheng et al., 1999; Stapleton et al., 2008, 2012; Jurkunas et al., 2009; Nielsen et al., 2015; Walther et al., 2017). Based on a perceived increase of ocular *Fusarium* isolates sent to the Dutch mycology reference laboratory (Center of Expertise in Mycology Radboudumc/CWZ, Nijmegen, the Netherlands) for identification and *in vitro* susceptibility testing, a national survey was initiated. As there was no surveillance or registry of keratitis cases in the Netherlands, we traced possible keratitis cases using the *Fusarium* cultures that had been sent to the mycology reference laboratory. Through this survey we aimed to describe the epidemiology of *Fusarium* keratitis, as well as the clinical implications and management.

## Materials and Methods

The culture collection of the Dutch mycology reference laboratory was searched for *Fusarium* isolates from corneal scrapings, corneal swabs and from contact lens (CL) fluid, which had been sent for identification and *in vitro* susceptibility testing between the beginning of 2005 and the end of 2016. Isolates were identified to the genus level using microscopic and macroscopic characteristics. *Fusarium* isolates from patients with confirmed fungal keratitis were identified to the species level through sequencing of the ITS1-5.8S-ITS2 region of the rDNA and TEF1 gene, as described previously (Salah et al., 2015).

*In vitro* antifungal susceptibility testing (AFST) was performed according to the EUCAST microdilution reference method (Roiquez Tudela et al., 2008; Arendrup et al., 2015). The antifungal agents tested included amphotericin B (Bristol Myers Squibb), voriconazole (Pfizer), and natamycin (Sigma-Aldrich). In addition, the activity of the antiseptic agent chlorhexidine (Pharmaline) was tested.

Through the clinical microbiology laboratories that had sent *Fusarium* isolates to the mycology reference laboratory, ophthalmologists were identified who had treated the keratitis infection. The ophthalmologists were asked to complete an online questionnaire regarding the keratitis case including demographic data (year of birth and gender) and clinical data including usage and type of CLs, visual acuity (VA) at diagnosis and after treatment, time between start of complaints and diagnosis of fungal keratitis and which kind of treatment was used including corticosteroid use. Antifungal treatment failure was defined as the need of corneal transplantation or enucleation/evisceration. Visual acuity was classified according to the ICD-11/“WHO classification of vision impairment” (ICD-11 for Mortality Morbidity Statistics, 2019). The categories of distance visual impairment are; mild (Snellen VA worse than 0.5), moderate (Snellen VA worse than 0.3), severe (Snellen VA worse than 0.1), and blindness (Snellen VA worse than 0.05 or only light perception).

The statistical analysis was performed with IBM SPSS Statistics 25. To estimate the association of CL wear with fungal keratitis the number of CL wearers in the Dutch community in 2012 served as control group (Bruggink, 2013). The collection of samples and clinical data from the patients were collected and processed in agreement with the Declaration of Helsinki.

## Results

### Epidemiology and Patient Demographics

**Figure 1:**
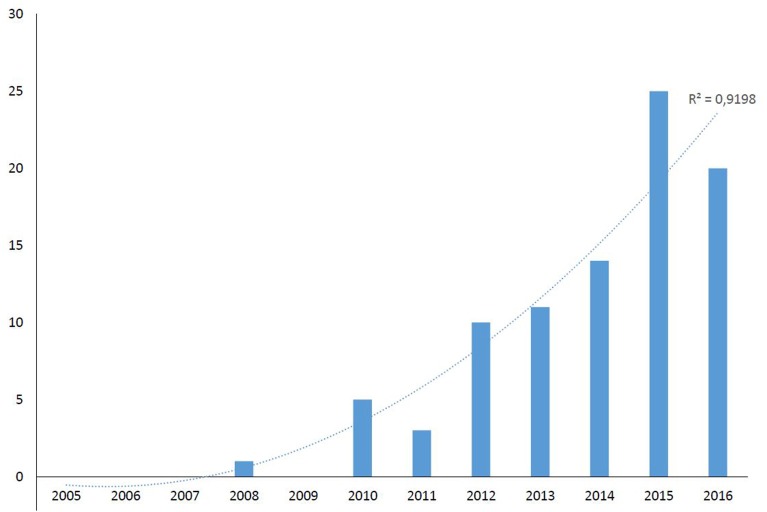
Number of *Fusarium* keratitis cases in The Netherlands during the period 2005−2016.

On the basis of collected isolates, 89 *Fusarium* keratitis cases were identified from 16 different hospitals in the Netherlands over a period of 12 years. Only one case was identified in the first 5 years (2005–2009), while all other cases were found from 2010 onwards (Figure 1). The number of cases of *Fusarium* keratitis showed a significant increasing trend over time (*R*^2^ = 0.9199). The population in the Netherlands was ~16.6 million in this period, which leads to a mean incidence of 0.45 (range 0–1.5) per million per year. The male (*n* = 32) to female (*n* = 57) ratio was 1:3 (*p* = 0.014) with a mean age of 42 years (range 13–85). Other clinical characteristics are shown in Table 1.

**Table 1:** Clinical characteristics and outcome in Dutch patients with *Fusarium* keratitis.

	**No. (% or range)**
**Total no. of cases**	89
**Age (years)**	42 (13–85)
**Gender (women)**	57 (64%)
**Contact lenses**	
Soft	65 (68.5%)
Rigid	4 (4.5%)
None	5 (5.6%)
Unknown	19 (21.4%)
**Trauma**	
Yes	5 (5.6%)
No	84 (94.4%)
**Visual acuity at diagnosis**^†^	
logMAR	0.8 (−0.10 to 3)
Snellen	0.16 (0.001–1.25)
**Visual acuity after treatment**^†^	
logMAR	0.2 (0.2–2)
Snellen	0.63 (0.01–0.63)
**Outcome**	
Cornea transplantation	20 (22.5%)
Enucleation/evisceration	7 (7.9%)
Healed infiltrate	62 (69.6%)
**Corticosteroids use before diagnosis**	
Yes	16 (18%)
No	50 (56.2%)
Unknown	23 (25.8%)
**Antimicrobial treatment at diagnosis**	
Antibiotics	39 (43.8%)
Antifungals	1 (1.1%)
Combinations^*^	25 (28.1%)
None	1 (1.1%)
Unknown	23 (25.8%)
**Antimicrobial treatment after diagnosis**	
Amphotericin B (T)	7 (7.9%)
Amphotericin B (T+S)	1 (1.1%)
Voriconazole (T)	14 (15.7%)
Voriconazole (T+S)	6 (6.7%)
Voriconazole (T+S+I)	1 (1.1%)
Natamycin (T)	1 (1.1%)
Chlorhexidine (T)	5 (5.6%)
Antibiotics	8 (9%)
Combinations^*^	21 (23.6%)
None	4 (4.5%)
Unknown	21 (23.6%)

* *Combinations of antibiotics, antifungals, antivirals, and/or antiseptics*. *Administration route: T, topical; S, systemic; I, intraocular*.

† *Mean (range)*.

### Treatment Strategies

In 66 cases the treatment was known at the time of diagnosis of fungal keratitis (one of which received no antimicrobial therapy), and treatment strategies varied greatly. All 65 patients received topically administered antimicrobial agents (Table 1). In 39 (59.1%) patients only topical antibiotics were used, while 25 (37.9%) patients received a combination of antibiotics, antivirals and/or antifungals. One patient was already receiving antifungal therapy (intraocular injected amphotericin B) when the diagnosis *Fusarium* keratitis was made. Of 68 cases the treatment strategy after a fungus was cultured was known (see Table 1 for more details). Therapy was changed to amphotericin B in eight patients (11.8%), to voriconazole in 21 patients (30.9%), and in one case to natamycin, which is not commercially available in the Netherlands. Eight patients continued with antibiotics alone. In 21 patients (30.9%) a combination of antibiotics, antivirals and/or antifungals was administered. Of note, five patients were treated with chlorhexidine 0.02% monotherapy after *Fusarium* was cultured.

### Treatment Outcome

Keratitis management was not successful in 27 (30%) patients, with 20 (22%) patients needing corneal transplantation. Three eyes, which already underwent a cornea transplant to debulk the fungal load, were eventually enucleated and evisceration was performed in four additional cases (8%). The mean VA was moderately impaired at the time of *Fusarium* keratitis diagnosis with a logMAR of 0.8 (95% CI 0.6–1, Snellen equivalent 0.16). The final VA outcome (excluding the enucleated/eviscerated patients) measured 0.2 logMAR on average (95% CI 0.1–0.3, Snellen equivalent 0.63), which is compatible with normal everyday activities (suboptimal but within the range of normal vision). Regarding the VA there was no significant difference between a severe or mild to moderate outcome regarding corticosteroid use before diagnosis (*p* = 0.08, Fisher's Exact Test). Complete response was achieved with chlorhexidine monotherapy in four of five patients, whereas one required a cornea transplantation.

### Risk Factors

Only five patients recalled a trauma to the affected eye; due to penetration by biological material or a corpus alienum. In 70 patients information regarding the use of CLs was available, and 65 (93%) of these wore a contact lens in the affected eye; primarily soft CLs (94%) and only a few rigid gas permeable CLs (6%). The time between start of symptoms and the diagnosis of fungal keratitis was significantly longer in the group with a poor outcome (VA worse than 0.1 logMAR, Snellen equivalent 0.8) as opposed to the group with (partially) restored vision; respectively, 22 vs. 15 days (*p* = 0.024, calculated by logistic regression and corrected for missing data). Corticosteroids were frequently used before the diagnosis of fungal keratitis was made. In this cohort 18% of the cases were given topical corticosteroids alongside antimicrobial agents.

### Strain Identification and Susceptibility Profile

Molecular species identification, showed that *F. oxysporum* (*n* = 22, 24.7%) was the most frequently isolated species followed by *F. solani sensu stricto* (*n* = 16, 18%) and *F. petroliphilum* (*n* = 8, 9%) (Table 2). Based on the assignment of the isolates to the according species complex, as described by Salah (Salah et al., 2015), the most frequent encountered complexes were *F. solani* species complex (FSSC, *n* = 32, 36%), followed by *F. oxysporum* species complex (FOSC, *n* = 22, 24.7%) and *F. fujikuroi* species complex (FFSC, *n* = 15, 16.9%). Overall, 10 isolates were not molecularly identified and one isolate could not be speciated and is believed to represent a new *Fusarium* species. A relationship between *Fusarium* species and disease severity of treatment outcome could not be established. *In vitro* susceptibility testing indicated that amphotericin B was the most active antifungal agent followed by natamycin, voriconazole, and chlorhexidine (Table 2).

**Table 2:** Molecularly identified fusarial keratitis isolates and their susceptibility profile including chlorhexidine (only identified strains are depicted, *n* = 78 and MICs presented as commonly used concentrations).

	MIC^*^ % [mode (range)]	MIC^*^ **mg/L [mode (range)]**				
***Fusarium* species complex and species (*n*)**	**CHX**	**CHX**	**AMB**	**VCZ**	**NAT**	**POS**
***F. solani*** **species complex—FSSC (32)**	0.003 (0.0015–0.006)	16 (8–32)	2 (0.5–16)	8 (4–16)	8 (4–16)	16
*F. solani* (16)	0.006 (0.0015–0.006)	32 (8–32)	2 (1–16)	8 (4–16)	8 (4–16)	16
*F. petroliphilum* (8)	0.0015 (0.0015–0.006)	8 (8–32)	2 (0.5–4)	8 (4–16)	4 (4–8)	16
*F. keratinoplasticum* (7)	0.003 (0.0015–0.006)	16 (8–32)	4 (2–4)	4 (4–16)	4 (4–8)	16
*F. falciforme* (1)	0.006	32	2	16	8	16
***F. oxysporum*** **species complex—FOSC (22)**	0.0015 (0.0002–0.013)	8 (1–64)	2 (0.25–16)	4 (2–16)	8 (4–8)	16
*F. oxysporum* (22)	0.0015 (0.0002–0.013)	8 (1–64)	2 (0.25–16)	4 (2–16)	8 (4–16)	16
***F. fujikuroi*** **species complex—FFSC (15)**	(0.0008–0.013)^#^	(4–64)^#^	2 (1–4)	4 (1–8)	8 (2–8)	16 (0.25–16)
*F. proliferatum* (7)	0.0008(0.0008–0.013)	4 (4–64)	1 (1–4)	4 (2–8)	8 (4–8)	(2–16)^#^
*F. verticillioides* (2)	(0.0008–0.003)^#^	(4–16)^#^	(1–2)^#^	(1–2)^#^	(2–8)^#^	(0.25–0.5)^#^
*F. lactis* (3)	0.0015 (0.0015–0.003)	8 (8–16)	(0.5–4)^#^	(2–8)^#^	8	16 (2–16)
*F. sacchari* (1)	0.0015	8	2	1	8	0.25
*F. ramigenum* (1)	0.003	16	4	1	4	1
*F. musae* (1)	0.003	16	2	4	8	1
***F. dimerum*** **species complex—FDSC (7)**	0.0015 (0.0015–0.003)	8 (8–16)	2 (0.5–2)	8	4 (4–16)	16
*F. dimerum* (7)	0.0015 (0.0015–0.003)	8 (8–16)	2 (0.5–2)	8	4 (4–16)	16
***F. equiseti-incarnatum*** **species complex—FIESC (1)**	^†^	^†^	1	8	^†^	16
*F. equiseti* (1)	^†^	^†^	1	8	^†^	16
***Ambrosia Fusarium*** **complex—AFC (1)**	0.006	32	2	16	8	16
*F.ambrosium* (1)	0.006	32	2	16	8	16

* *MIC, minimal inhibitory concentration; AMB, amphotericin B; VCZ, voriconazole; POS, posaconazole; NAT, natamycin; CHX, chlorhexidine*.

# *Mode not calculable (even numbers)*.

† *Susceptibility testing was not performed*.

## Discussion

Our retrospective study of *Fusarium* cultures obtained from cornea samples and subsequent case finding indicated a significant increase of *Fusarium* keratitis cases in the Netherlands since 2010. We found an estimated mean incidence of 0.45 cases per million per year, which increased from sporadic cases in 2005 to 1.5 cases per million in 2015. We acknowledge that our estimate is prone to bias as we lack a reliable denominator and practices of sending fungal isolates to the mycology reference laboratory might have changed over time. However, our observation is in keeping with a general increase of keratitis cases and reports of increasing fungal keratitis cases in other countries such as the USA, Denmark and Germany (Jurkunas et al., 2009; Nielsen et al., 2015; Walther et al., 2017). Furthermore, one previous study indicated that the incidence of fungal keratitis was very low before 2010 in the Netherlands (Cheng et al., 1999). In 1996, 92 cases of microbial keratitis were identified through a 3-month national survey in the Netherlands, with no cases due to *Fusarium* species (Cheng et al., 1999). A second single center study identified 109 keratitis cases over a 5-year period (2005–2009) that required hospitalization (Hoddenbach et al., 2014). Two cases of fungal keratitis were identified (1.8%), which may indicate that the course of infection is relatively severe in fungal infection compared with bacterial or viral infection.

Our survey indeed indicated that *Fusarium* keratitis is a severe infection with almost one-third of patients failing initial therapy. There are several factors that contribute to a poor outcome including delayed diagnosis. We could not assess if delay was due to patients delay, physicians delay or a combination of both. However, all patients who required evisceration or enucleation were diagnosed more than 14 days after onset of symptoms, underscoring that early diagnosis (and treatment) is critical for a favorable outcome. Early diagnosis could involve adding fungal culture media if cornea scrapings are sent for culture, or adding fungal targets to molecular testing. Another factor is the use of corticosteroids, which is a known risk factor for invasive fungal diseases and mycotic keratitis (Thomas and Kaliamurthy, 2013). In our cohort 18% of the patients received topical corticosteroids before the diagnosis of fungal keratitis. Unfortunately, the data set did not contain the used dosage of corticosteroids which presents a limitation of our study. We could, however, not demonstrate a significant difference between mild to moderate and severe outcome and the use of corticosteroids.

Given the wide variety of treatment strategies, our survey indicated an apparent uncertainty how best to treat *Fusarium* keratitis. Although the national guideline recommends topical amphotericin B (Kullberg et al., 2017), most regimens used in our cohort included voriconazole. The ocular toxicity and symptoms associated with local amphotericin B in combination with the well-documented ocular penetration of voriconazole 1% eye drops may have played a role in this transition. One problem is a lack of convincing studies to support evidence-based treatment choices, as current clinical trials are generally underpowered and of variable quality (FlorCruz and Evans, 2015). Furthermore, the available antifungal agents including amphotericin B 0.15% and voriconazole 1% are not commercially available as ophthalmic drops in our country and are made-to-order by hospital pharmacists.

Rational treatment choices are also hampered by the lack of correlation between MIC and drug efficacy. The most frequently isolated *Fusarium* species complexes (FSSC, FOSC and FFSC) are generally multidrug resistant *in vitro* and any activity is at best fungistatic (Oliveira dos Santos et al., 2019). The use of MICs to guide treatment choices is limited due to the fact that through topical therapy the fungus is exposed to very high drug concentrations and thus may respond despite a high *in vitro* MIC. There is increasing evidence that chlorhexidine may be effective for the treatment of *Fusarium* keratitis, supported by both clinical trials and *in vitro* studies (Rahman et al., 1997, 1998). We recently showed that chlorhexidine exhibited fungicidal activity against a broad range of filamentous fungi including *Fusarium* species, at concentrations that could be readily achieved clinically (Oliveira dos Santos et al., 2019). The activity of chlorhexidine against *Acanthamoeba* and bacteria makes it a very attractive agent for local treatment of keratitis. However, not much is known about its penetration into the cornea and aqueous humor. Theoretically, chlorhexidine digluconate should penetrate the cornea better than the polyenes which have a higher molecular weight.

The main risk factor for *Fusarium* keratitis was the use of soft CLs for extended wear. In our cohort with known reported use or non-use of CLs, 93% (73% of the complete cohort) of the cases wore CLs, this is much higher than the normal coverage of CL use in the normal Dutch population. In 2012 the prevalence of CL wearers in the Dutch population was estimated to be 12% (Bruggink, 2013). In the years prior to and after 2012 the prevalence did not fluctuate significantly. The odds of contracting fungal keratitis amongst CL wearers is therefore 20 times higher than in non-CL wearers (OR 19.8, 95% CI: 9.39–41.87). Stapleton et al., also found a high incidence of microbial keratitis in wearers of different CL types in comparison to the community, but the study did not report fungal keratitis cases (Stapleton et al., 2008, 2012). We can only speculate on the cause of the observed increasing frequency of *Fusarium* keratitis. Possibilities include (1) non-compliance to the approved maximum wear recommendation of extended wear CLs, (2) cleaning CLs with tap water or saliva, and (3) reduced fungal activity of CL solutions. Prevention of infection through education on proper use of CLs and adherence to recommended cleaning and disinfection protocols remains an important goal.

Although *Fusarium* keratitis remains a rare complication of CL wear, our study indicates that the course of infection may be severe, diagnosis is often delayed and treatment options are limited. Systematic surveillance is needed to confirm our findings and monitor trends in prevalence. Furthermore, a consensus treatment strategy is warranted to optimize and standardize antifungal therapy. The antiseptic chlorhexidine might be an attractive treatment option given its broad antimicrobial activity but further studies would be required to confirm its efficacy and safety.

## Data Availability Statement

The datasets generated for this study are available on request to the corresponding author.

## Ethics Statement

Ethical review and approval was not required for the study on human participants and their collected samples in accordance with the local legislation and institutional requirements. Written informed consent from the patients was not required to participate in this study in accordance with the national legislation and the institutional requirements.

## Author Contributions

CO, EK, CE, and PV contributed to conception and design of the study. EK and CO organized the database. CO performed the statistical analysis and wrote the first draft of the manuscript. JR, RS, NV, YC, and NS provided the ophthalmologic data. All authors contributed to manuscript revision, read, and approved the submitted version.

### Conflict of Interest

PV reports grants from Gilead Sciences, MSD, Pfizer, F2G, and non-financial support from OLM and IMMY, outside the submitted work. The remaining authors declare that the research was conducted in the absence of any commercial or financial relationships that could be construed as a potential conflict of interest. The reviewer AW declared past co-authorship with one of the authors PV to the handling editor.
